# Thermal, Rheological, and Moisture Absorption Behaviours of Polyvinyl Alcohol (PVA)/Lignin Composites

**DOI:** 10.3390/polym17212918

**Published:** 2025-10-31

**Authors:** Erdem Selver, Ayca Dogrul Selver, Abu Saifullah, Zhongyi Zhang, Hom N. Dhakal

**Affiliations:** 1 Portsmouth Centre for Advanced Materials and Manufacturing (PCAMM), School of Electrical and Mechanical Engineering, University of Portsmouth, Portsmouth PO1 3DJ, UK; erdem.selver@port.ac.uk (E.S.); abu.saifullah@port.ac.uk (A.S.); zhongyi.zhang@port.ac.uk (Z.Z.); 2 Department of Textile Engineering, Kahramanmaras Sutcu Imam University, Kahramanmaras 46100, Turkey; 3 Department of Geological Engineering, Kahramanmaras Sutcu Imam University, Kahramanmaras 46100, Turkey; ayca.dogrul@gmail.com

**Keywords:** polyvinyl alcohol (PVA), lignin, thermal properties, water absorption, rheology

## Abstract

Lignin and polyvinyl alcohol (PVA) polymer have both been used as biodegradable materials for many years, enabling the development of eco-friendly composite structures. In this study, a PVA polymer was blended with different proportions of lignin ranging from 0.5 wt% to 10 wt% and their thermal, rheological and moisture absorption behaviours were analysed and compared. According to rheology tests, addition of lignin decreased the viscosity of PVA up to 25% by creating plasticization effect. Thermal tests reveal that the addition of lignin had no significant effect on the melting behaviour of the PVA polymer. However, the amount of char residue increased from 0.48% to 4.15% as the lignin content increased to 10 wt%, indicating improved thermal stability of the PVA polymer. The hydrophobic nature of lignin particles helped to reduce the moisture absorption of PVA polymers up to 6% especially at high wt% lignin loadings.

## 1. Introduction

Poly (vinyl alcohol) (PVA) is a biodegradable synthetic polymer with unique features such as non-toxicity, excellent film-forming ability, high transparency, and superior oxygen barrier performance [1,2,3]. Its high density of hydroxyl groups, however, results in strong hydrophilicity, leading to excessive water absorption and poor moisture and vapour resistance [4,5,6]. These limitations hinder its use in fields such as food packaging, biomedical materials, and coatings. Considerable research has therefore focused on strategies to improve the water resistance, barrier efficiency, and thermal stability of PVA for practical applications [7,8,9,10].

Lignin, a major component of lignocellulosic biomass, is abundantly available as a byproduct of the pulp and paper industry. Once considered a low-value residue, lignin has attracted significant attention as a sustainable and low-cost feedstock for polymer composites due to its carbon neutrality, aromatic structure, and diverse functional groups [11]. Its hydroxyl, carboxyl, and methoxy groups allow strong interactions with polymer matrices, while its hydrophobic character contributes to barrier and stability improvements [12,13,14,15]. Recent research has highlighted lignin’s potential to enhance biopolymers, both as a reinforcing filler and as a functional additive, thereby promoting high-value lignin-based materials [16,17,18]. Research on lignin–PVA composites can be broadly classified into the following four categories:

*Interfacial interactions and compatibility*—Lignin is a cheap, sustainable, and widely accessible polymeric material that interacts strongly with PVA due to the abundance of hydroxyl groups in both polymers. These interactions, mainly hydrogen bonding between lignin’s hydroxyl, carboxyl, and methoxy groups and the hydroxyl groups of PVA, can enhance interfacial adhesion, restrict chain motion, and improve thermal stability [19,20,21,22,23].

*Moisture and barrier properties*—The incorporation of lignin has been shown to decrease water vapour transmission rates, increase surface hydrophobicity, and enhance ultraviolet resistance. For example, lignin nanoparticles and functionalized lignin improved water repellence and UV shielding when blended with PVA [24,25,26,27,28].

*Mechanical and thermal performance*—Several studies have demonstrated that adding small amounts of lignin significantly improves the tensile strength, modulus, and thermal stability of PVA. These effects are generally attributed to strong hydrogen bonding and restricted chain mobility between lignin and [29,30,31,32,33,34,35].

*Rheological behaviour*—Literature reports are more varied. Some studies observed that lignin addition reduces viscosity and storage modulus, suggesting a plasticizing or chain disentanglement effect [36,37,38,39,40]. Others, however, reported viscosity increases at higher lignin loadings, likely due to hydrogen bonding and limited chain motion [41,42,43].

Taken together, these results confirm that lignin can beneficially modify the properties of PVA. At the same time, the diversity of lignin types, concentrations, and processing methods has led to inconsistent findings.

Although many published works highlight the positive effects of lignin on PVA, most focus on individual properties, rely on high lignin loadings, or employ chemically modified lignin. For instance, several works examined thermal and mechanical reinforcement at relatively high lignin loadings (typically > 10 wt%), often emphasising enhanced tensile strength, modulus, and thermal decomposition temperature [44,45,46]. Additional research on barrier performance revealed that lignin nanoparticles or chemically modified lignins greatly enhance UV resistance and decrease water vapour penetration [47,48]. These advancements, however, frequently come at the cost of optical transparency or flexibility, which restricts their usefulness in coating or packaging applications. There are currently few systematic studies reported on how lignin influences moisture resistance, thermal stability, and rheological behaviour all at once, especially at low lignin concentrations [49,50]. In order to increase compatibility with PVA, the majority of earlier studies also used chemically modified lignins, such as acetylated, grafted, or sulfonated versions; nevertheless, there are still a few studies using unmodified lignin at low concentrations (<5 wt%) [29,51]. Such low-load systems are particularly important because they can preserve the inherent film-forming and optical properties of PVA while achieving balanced performance enhancement [26]. In this study, the influence of lignin loading (0.5–10 wt%) on the thermal, rheological, and moisture properties of PVA is systematically investigated. By evaluating these functional properties together, this work provides new insights into the role of lignin concentration in tailoring PVA composites and supports the development of sustainable materials for packaging and fibre-reinforced applications.

## 2. Experimental

### 2.1. Materials

Hydrophobically modified PVA (Exceval-AQ 4104, Kuraray, Japan) is supplied from Kuraray. The molecular weight of PVA is 48,400 (g·mol ^−1^). This grade is a hydrophobically modified PVA, containing acetate-based side groups, which are designed to reduce water sensitivity and enhance compatibility in blends.

Lignin was extracted from wheat straw using the organosolv procedure, which was generously provided by Fortum in Espoo, Finland. It has a molecular weight of Mw-3650, Mn-1620, and a polydispersity index (PDI) of 2.3. The wheat straw lignin used in this study is known to contain both syringyl (S) and guaiacyl (G) units. While the precise S/G ratio for this batch was not measured, literature reports for organosolv wheat straw lignin indicate an S/G ratio of approximately 0.6–0.9 [52,53,54]. The lignin exhibited a relatively high polydispersity index (PDI = 2.3), reflecting its heterogeneous molecular structure.

### 2.2. Manufacturing of PVA/Lignin Films

**Figure 1:**
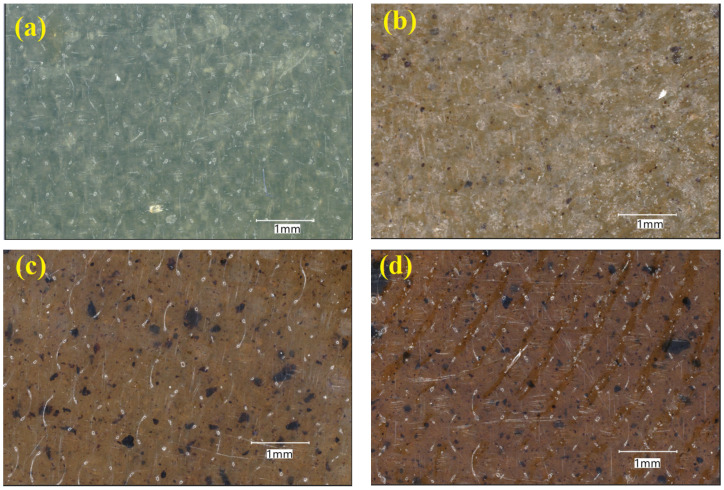
Microscopic images of: (**a**) PVA, (**b**) PVA-0.5L, (**c**) PVA-1.0L, and (**d**) PVA-1.5L.

PVA pellets were ground to 250 µ size using a Retsch ZM300 (Retsch, Haan, Germany) grinding machine at around 8500 RPM before being mixed with lignin particles to achieve a more uniform distribution. During the grinding operation, liquid nitrogen was employed to keep the temperature below 20 °C and prevent polymer sticking or softening. After obtaining fine PVA particles, they were combined with lignin powder in various ratios (0.5, 1, and 1.5 wt%) using a mechanical stirrer. Table 1 shows the sample codes and lignin content of the mixtures. PVA/lignin blends were hot-pressed using a JRD Bipel (Walsall, UK) lab-type compression moulder at 215 °C and 5 bars of pressure for 10 min, resulting in about 1 mm-thick PVA and PVA/lignin films as shown in Figure 1. Only for TGA tests, in addition to lower lignin concentration samples (0.5–1.5 wt%), higher lignin content samples (3%, 5% and 10%) were also prepared.

Figure 1 shows optical microscope images of PVA/lignin films with varying lignin loadings. The neat PVA film (Figure 1a) had a smooth and homogenous surface with no obvious phase separation, indicating excellent film-forming capabilities. Following the incorporation of 0.5 wt% lignin (Figure 1b), tiny dark domains corresponding to lignin particles were seen scattered throughout the PVA matrix. These domains were rather fine and equally distributed, indicating good compatibility with low lignin concentration.

At 1.0 wt% lignin (Figure 1c), the number and size of lignin domains increased. The darker aggregates show partial lignin clustering, which can function as localised stress concentrators and may alter rheological properties. The background matrix also seemed rougher than pure PVA, indicating reduced miscibility. At the highest loading tested (1.5 wt%), substantial aggregation was seen, with large and irregular lignin-rich areas scattered throughout the PVA phase. The presence of such aggregates demonstrates that miscibility diminishes with increasing lignin concentration, which is consistent with rheological studies indicating nonlinear effects at larger filler loadings.

Overall, the morphological evidence supports the interpretation that low lignin contents promote reasonably uniform dispersion within PVA, whereas higher loadings lead to visible aggregation and phase separation.

### 2.3. Characterisation

The differential scanning calorimetry (DSC) analysis was carried out using TA Q80 equipment (Waters Corporation, New Castle, DE, USA) to measure melting point and glass transition temperatures of PVA/lignin blends using ISO 11357-1:2023 standard [55]. The specimens were analysed under flowing nitrogen for three cycles of heating and cooling. The sample was first heated at a rate of 10 °C/min until it reached 260 °C. Then, it was cooled to 20 °C using a 10 °C/min cooling ramp. Finally, the temperature rose to 260 °C at a rate of 10 °C per minute. The crystallinity degree (X_c_) was calculated using Equation (1):

(1) $$Xc\left(\%\right)=\frac{{\Delta H}_{M}}{{\Delta H}_{M0}}\left(1-m_{f}\right)\times 100$$

where ΔH_M_ is the melting enthalpy, ΔH_M0_ is the melting enthalpy of 100% crystalline PVA which is 161.6 J/g, and (1 − m_f_) is the mass fraction of PVA [56].

Thermogravimetric analysis (TGA) was conducted utilising TA Q50 (Waters Corporation, New Castle, DE, USA) equipment to measure the quantity and rate of change in a sample’s mass as a function of temperature and time in a controlled environment using ISO 11358-1:2022 standard [57]. The samples were heated at 20 °C per minute until they reached 700 °C. Higher lignin loadings (3, 5, and 10 wt%) were included in the TGA experiments to specifically evaluate their effect on thermal stability, since lignin is known to act as a char-forming agent at elevated temperatures and can substantially alter the decomposition profile even at higher concentrations.

The composite films were subjected to two different environmental conditions (75% humidity-75 °C temperature and 85% humidity-85 °C temperature) to investigate their moisture absorption behaviours using the IEC 60068-2 test standard [58].

The moisture absorption (%) was calculated using Equation (2). Where M_1_ and M_0_ are the weight of the samples after and before exposing the humidity, respectively, as shown in Equation (2).

(2) $$Moisture\;Absorption\left(\%\right)=\frac{M_{1}-M_{0}}{M_{0}}\times 100$$

PVA and PVA/lignin films were rheologically characterised using a Discovery Hybrid Rheometer (TA Instrument, Waters Corporation, New Castle, Delaware, USA) in parallel plate geometry, with a 25 mm plate on the top and bottom and a 1000-micron gap in between, at 230 °C via ISO 3219-1:2021 test standard [59]. Flow-sweep rheological properties were measured at shear rates (˙γ) between 0.01 and 100 1/s. Dynamic strain-sweep experiments were performed between 0.01 and 100%. For both the flow-sweep and strain-sweep tests, data were collected at five points per decade.

An optical contact angle and surface tension metre CAM 101 (KSV Instruments, Helsinki, Finland) was used to measure the contact angles of PVA and PVA/lignin blends by placing a drop of deionised water onto the film surface. An average of five specimens was recorded and compared.

## 3. Results and Discussions

### 3.1. Rheological Test Results

**Figure 2:**
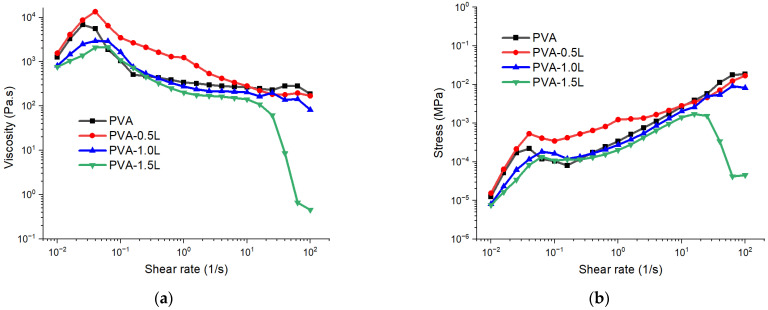
Flow-sweep plots of: (**a**) viscosity-shear rate and (**b**) stress-shear rate for PVA and PVA/lignin samples.

The flow-sweep test results for PVA and PVA/lignin samples at various shear rates are shown in Figure 2. PVA or PVA/lignin samples display shear-thinning behaviour (viscosity decreases with increasing shear rate), typical for polymer solutions and nano composites. The relation between apparent viscosity (η) and shear rate (˙γ) for a shear rate of 0.01–100 1/s is depicted in Figure 2a.

At low shear rates (10^−2^ to 10^−1^ 1/s), the viscosity of PVA is somewhat less than that of the samples containing 0.5% lignin, suggesting that small lignin additions strongly enhance network interactions and structuring at low shear rates. PVA-1.5L has the lowest viscosity, indicating possible disruption of the polymer network at higher lignin loadings while PVA-1.0 has the medium viscosity values. It is also clear that the viscosity of all samples increases until a shear rate of 0.1 is achieved. The viscosity then begins to decline substantially as a result of the polymers’ shear-thinning effect at high shear rates.

At the mid shear rate (~10^0^–10^1^ 1/s), PVA and low lignin blends (0.5L, 1.0L) maintain high viscosity plateaus, suggesting stable entanglement or network structures under moderate shear. PVA-1.5L drops faster here, hinting at weaker entanglement or possible phase separation.

At high shear rates (>10^1^ 1/s), neat PVA has the highest viscosity value compared to blends. It is also worth noting that the PVA-1.5L sample had a significantly lower shear rate, between 10 and 100, than the other samples. This means the network collapses entirely under high shear—possibly due to lignin aggregation or poor interfacial bonding. The viscosities of PVA, PVA-0.5L, PVA-1.0L, and PVA-1.5L are approximately 186, 169, 82, and 0.5 Pa·s, respectively, at the highest shear rate. The plasticisation effect of the lignin particles is more obvious when the shear rate is higher. Possari et al. [40] noticed a comparable drop in viscosity as the lignin ratio rose from 2.5% to 15%. Similarly, Long et al. [60] observed that the viscosity of the PLA matrix was reduced by adding 0.5% lignin powders. Additionally, the entanglement or interaction of polymer chains during the melting stage may be reduced by the presence of lignin particles. Figure 2b shows similar behaviour, with plain PVA having the largest shear stress compared to mixes where the shear rate is maximum.

**Figure 3:**
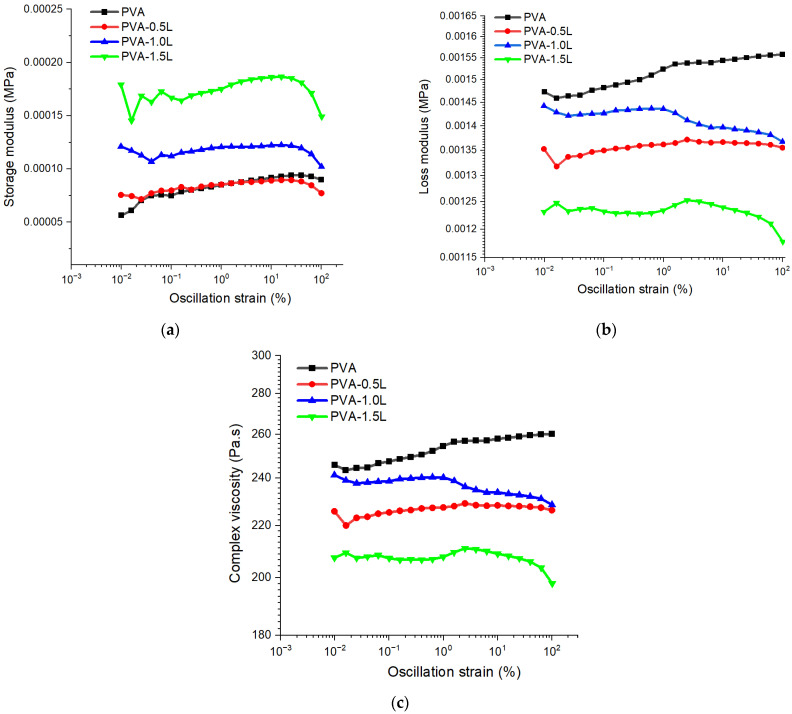
Dynamic strain-sweep plots of: (**a**) complex viscosity, (**b**) loss modulus and (**c**) storage modulus for PVA and PVA/lignin samples at strain range of 0.1–100%.

Dynamic strain-sweep plots in Figure 3a demonstrate that the inclusion of lignin particles increased the storage modulus (G′) of PVA at various strain rates (%). The highest G′ values are achieved when the lignin content is greatest. At the linear viscoelastic range (~10^−3^% to ~1% oscillation strain), G′ is virtually constant (flat plateau), indicating that the material’s internal structure is intact and responds elastically without disruption. The magnitude of G′ increases with lignin loading, indicating that lignin improves network stiffness. The modulus deviates in the nonlinear area, which ranges from ~1% strain to 100+%. G′ gradually reduces in pure PVA and PVA-0.5L (network softening and increased chain mobility). For PVA-1.0L and PVA-1.5L, G′ initially remains higher and even exhibits minor fluctuations/bumps, which are often caused by filler–polymer interactions that resist breakdown, followed by partial structural rupture at very high strains (>10–30%). Possible nonlinear effects for PVA-1.5L, including variations at high strains, could imply filler agglomeration, microphase separation, or slippage at polymer–filler interfaces. Optical microscopy (Figure 1) revealed lignin micro-aggregates, which support the interpretation of nonlinear rheological behaviour observed at higher strains. These micro-aggregates likely disrupt the homogeneous stress transfer and explain the irregularities noted in viscosity and storage modulus.

In contrast to the storage modulus (G′), the loss modulus (G″) decreased when the lignin content increased according to Figure 3b. It is well known that the viscous component is represented by the loss modulus (G″), whereas the elastic component is characterised by the storage modulus (G′). The neat PVA is more viscous than elastic (high G′′, low G′ in Figure 3a). This shows a weak network creation and simple chain motion. The addition of lignin reduces the loss modulus, resulting in restricted chain motion and reduced viscous damping. At 1.0% lignin loading, the balance of viscous and elastic responses indicates partial reinforcing but also potential heterogeneity. For 1.5% lignin loading, G′′ is the lowest, indicating that chain mobility is strongly reduced and the material’s behaviour is more elastic. It appears that lignin reduces the viscosity of PVA, making the composite more elastic. This impact is greater as lignin content increases, with 1.5% lignin demonstrating the greatest inhibition of energy dissipation.

The complex viscosity in Figure 3c generated identical results as the apparent viscosity; the lignin particles reduced the viscosity of the neat PVA up to 25% at the highest oscillation strain (%). The neat PVA had the maximum viscosity, confirming its dominant viscous behaviour. Lignin addition reduces viscosity, making the system less resistant to flow, more elastic, and less dissipative. There is some reinforcement but also chain disruption when the lignin level is low (0.5–1%). High lignin (1.5%) content results in a considerable drop in viscosity, most likely due to filler aggregation or poor dispersion, lowering effective stress transfer. Overall, increasing lignin content reduces the viscous contribution and transitions the composite to a more solid-like yet lower-viscosity system. Optical microscopy (Figure 1) of PVA/lignin composites confirmed that at 1.5 wt% loading, lignin domains formed visible micro-aggregates, supporting the interpretation that filler clustering reduced viscosity at high shear rates. Optimal lignin (~1%) displays balanced viscosity, lower than pure PVA but stable, indicating good filler–matrix interaction. Excess lignin (1.5%) considerably decreases viscosity, implying filler aggregation or weak interfacial adhesion, which facilitates deformation [37]. This reduced viscosity will be useful in the manufacturing of fibre-reinforced composites containing PVA polymers and lignin.

The rheological response of the composites may vary from batch to batch due to the heterogeneity of lignin and its comparatively high PDI. While it is anticipated that the broad trends in viscosity and modulus will not change, slight changes in the composition of lignin (such as the S/G ratio or the distribution of molecular weight) may cause absolute values to change. PVA’s molecular weight distribution and surface modification are anticipated to influence its lignin miscibility and rheological response. Hydrophobic side groups may promote hydrophobic interactions with lignin, while preventing excessive hydrogen bonding between them, leading to an overall enhanced interaction, and increased processability and maintenance of filler–matrix interactions.

The observed shear-thinning behaviour, notably the viscosity decreasing at higher strain rates (Figure 2a), is advantageous for traditional melt-processing processes such as extrusion and injection moulding, where reduced viscosity promotes polymer flow and reduces energy consumption. Higher loadings of lignin micro-aggregates (≥1.5 wt%) may cause die swell, flow instabilities, and surface defects during film casting. In practice, this means that while modest lignin additions increase processability, higher loadings may necessitate compatibilizers, dispersants, or lignin modifications (such as acetylation or esterification) to maintain stable extrusion behaviour and homogeneous film shape. Such factors are critical when scaling laboratory results to industrial applications, as both flow stability and film quality directly affect end-of-life performance.

### 3.2. Thermal Test Results

**Figure 4:**
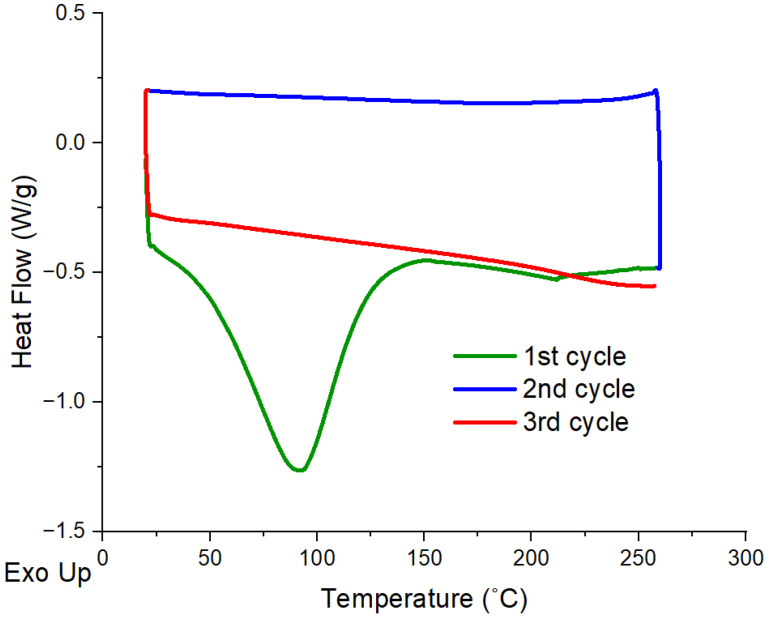
DSC curves of lignin.

**Figure 5:**
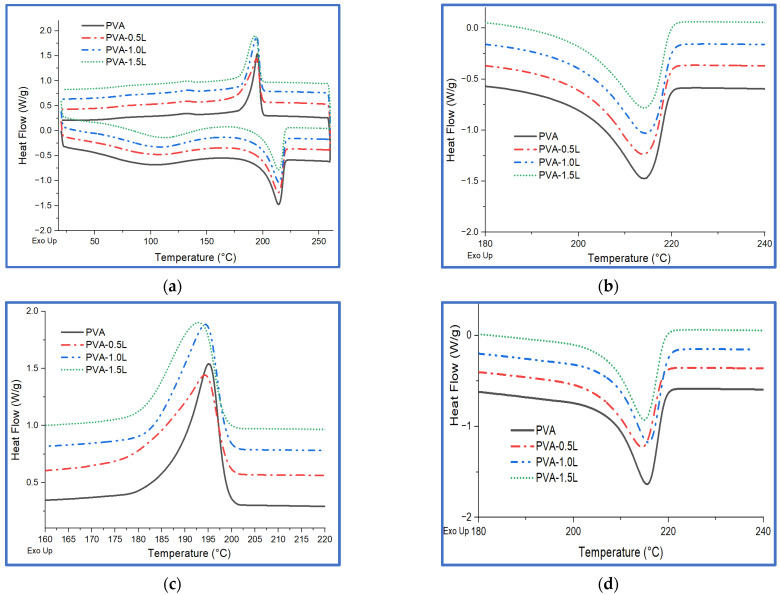
DSC curves of PVA and PVA/lignin blends: (**a**) 1st heating and cooling cycle together, (**b**) 1st heating cycle, (**c**) cooling cycle, and (**d**) 2nd heating cycle.

The DSC results of lignin, PVA and PVA/lignin blends are shown in Table 2 and Figure 4 and Figure 5 to assess the melting temperature (Tm), crystallisation temperature (Tc), and degree of crystallinity (Xc) of the samples under the various heating and cooling cycles specified in the experimental section.

Figure 4 presents DSC curves of lignin particles for all heating and cooling cycles. The first heating cycle shows a sharp peak at around 100 °C due to the removal of moistures already inside the lignin particles. However, this peak disappears at the second heating cycle (3rd cycle) as expected. Although some of the works in the literature indicate that the glass transition temperature (Tg) of lignin is between 50 and 180 °C [61,62,63,64], it is quite difficult to observe a sharp peak to calculate the Tg of the lignin for this study.

Table 2 shows that the inclusion of lignin did not affect the melting points of PVA during the first heating cycle measurement. Korbag et al. [65] observed only minor changes in the PVA melting peak while crystallinity was altered when a different lignin loading was applied (up to 66%). This first heating cycle (Figure 5b) is primarily used to reset the polymer’s thermal history while also eliminating moisture from the polymer. The nucleating effect of the lignin fillers creates a modest increase in crystallinity (Xc) after 0.5% and 1% at this cycle. During the cooling stage (Figure 5c), the crystallisation temperature (Tc) reduced slightly, particularly at 1.5% lignin loading (PVA 195.07 °C, 0.5L 194.31 °C, 1.0L 194.36 °C, 1.5L 192.96 °C). These lower temperatures of crystallisation suggest that lignin hinders the process, most likely as a result of chain–lignin interactions and/or steric hindrance of PVA chain diffusion [66]. Also, this could be attributed to the heterogeneous structure of the mixture by the addition of a large amount of lignin.

The second heating cycle (Figure 5d) results in increased melting points for PVA and PVA/blends. However, all samples showed a decrease in crystallinity as compared to the first heating cycle. Also, Xc is lower in all PVA/lignin blends vs. neat PVA, notably at 1.0L (46.31% → 43.01%) at the 2nd heating cycle. This is the cleaner indication of lignin reducing the recrystallization ability of the PVA polymer. This could be owing to entanglements between PVA chains and lignin, which prevents polymer chain folding and crystal formation [56]. The melting point of PVA decreased somewhat during the second heating cycle with 0.5% and 1.5% lignin loading. This is because chains begin to fracture at lower temperatures, releasing volatile portions of the lignin [67]. The DSC results reveal that the inclusion of lignin had no significant effect on the melting behaviour of the PVA polymer, which is primarily beneficial for the usage of PVA/lignin blends as a matrix part of fibre-reinforced thermoplastic composites. Similar results were observed by Siregar et al. [68] while the melting point and the crystallisation temperatures remained very similar when 5 to 20% lignin was added to the PVA polymer.

**Figure 6:**
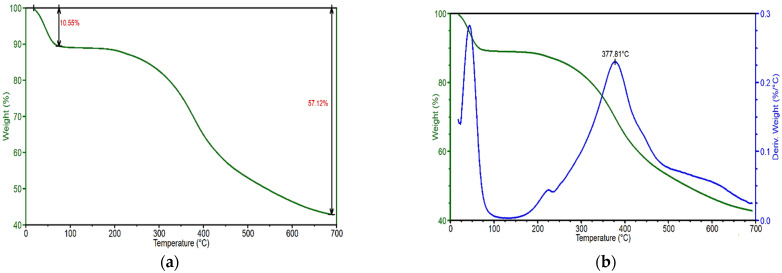
(**a**) TGA and (**b**) DTGA analyses of lignin.

Figure 6 shows the thermogravimetric measurement of lignin particles at various phases of mass loss. The first mass loss (10.55%) is attributed to moisture evaporation at roughly 90 °C, as illustrated in Figure 6a. After the first weight loss, the degradation process is slowed between 100 and 200 °C. The second mass loss begins at around 225 °C as lignin decomposes to volatile water, carbon dioxide, and char, and continues until 400 °C (Figure 6b). According to derivative thermal gravimetric analyses (DTGA), lignin pyrolysis begins around 378 °C, as illustrated in Figure 6b. This temperature (378 °C) also represents the temperature at which the highest rates of weight change (decomposition temperature) for lignin occur. At 700 °C, more than 40% of lignin samples survive due to the formation of extremely condensed aromatic structures.

**Figure 7:**
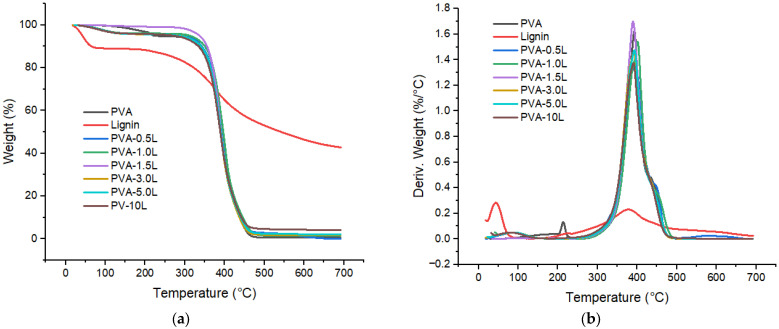
(**a**) TGA and (**b**) DTGA curves for PVA, lignin and PVA/lignin blends.

Table 3 and Figure 7 compare the decomposition temperature (Td) and char ratios for PVA and other samples including 3%, 5% and 10% of lignin loadings at 700 °C. Decomposition onset temperature of pure PVA is around 351 °C with the pyrolysis starting at around 393 °C. It is observed that the rate of the weight loss of neat lignin due to thermal degradation is lower than that of the PVA, as shown in Figure 7a. The derivative curve of PVA is exhibited at a higher temperature than that of lignin; it is 393 °C for PVA and 378 °C for lignin as presented in Table 3 and Figure 7b.

Table 3 shows that adding lignin slightly enhanced the onset degradation temperature of the pure PVA polymer. However, as lignin content increases, onset temperatures do not rise in direct proportion. For example, the sample containing 1% lignin (PVA-1.0L) has the greatest onset temperature. This could be due to reducing the molecular interaction between PVA and lignin by increasing lignin concentration. It is obvious that 10% lignin did not affect the onset temperature at all. This is again owing to the large amount of lignin, which caused agglomeration and decreased interaction with PVA. Similar enhanced initial resistance and maximum stability results were observed by De Freitas et al. [69] after using lignin powder to create PVA/lignin films.

When comparing DTGA peaks (Figure 7b) and decomposition temperatures, samples containing 1% and 1.5% lignin had higher values than pure PVA. Cao et al. [51] observed comparable thermal behaviour, with the breakdown temperature of PVA increasing from 258.6 °C to 262.5 °C when 10% lignin nanoparticles were added. Similarly, Xu et al. [33] found that applying 15% lignin loading raised the decomposition temperature of PVA by 5 °C. The other PVA/lignin samples had slightly lower decomposition temperatures than PVA, as shown in Table 3 and Figure 7b. This is achievable due to the addition of lignin, which has a lower decomposition temperature (378 °C), further lowering the breakdown temperature of PVA/lignin mixtures. The values decrease as the lignin content increases. The degradant lignin phenolic hydroxyl and carboxylic groups are also responsible for this [70].

Table 3 also shows the total amount of residues after TGA tests for all samples. Pure lignin powders have a remaining residue of around 43%. However, during the TGA experiments, the PVA polymer almost completely decomposed at 700 °C. When comparing all samples, the amount of residue increases as the lignin content increases, with the 10% lignin sample (PVA-10L) having the greatest char residue at roughly 4%. This is because, as Figure 6 and Figure 7 demonstrate, lignin is far more thermally stable than PVA at the test temperature.

### 3.3. Humidity and Contact Angle Test Results

**Figure 8:**
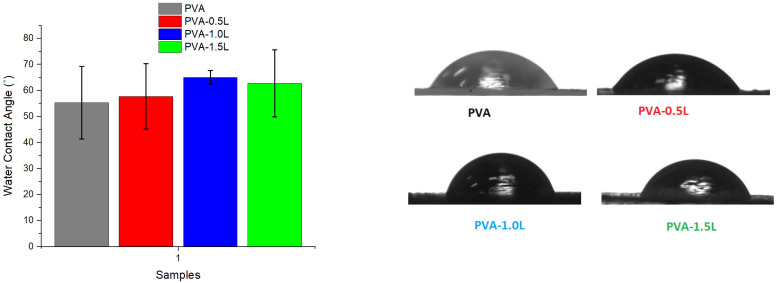
Water contact angle values and images of PVA and PVA/blends.

Water contact angle (WCA) measurements were used to assess the surface wettability of PVA and lignin/PVA composites (Figure 8). Because of the large number of hydroxyl groups throughout the polymer backbone, neat PVA has a contact angle of around 55°, which is consistent with its very hydrophilic nature. WCA increased somewhat when lignin was added at 0.5–1.5 weight percent; the greatest improvement was seen at 1.0 weight percent (~64°). This implies that lignin decreased PVA’s surface hydrophilicity, most likely via two mechanisms: (i) hydrogen bonding between the two molecules, which decreases the availability of some hydroxyl groups for water interaction; and (ii) partial surface segregation of lignin’s aromatic domains, which are more hydrophobic and less polar. Rynkowska et al. [71] achieved comparable contact angle test results, while the inclusion of nano additives increased the contact angle from 86 to 92 degrees.

At higher loadings (1.5 wt%), WCA was slightly reduced compared to the 1.0 wt% sample, although it remained greater than pure PVA. This non-monotonic behaviour could be due to changes in dispersion. At low loadings, lignin is evenly distributed and contributes mostly to hydrogen bonding and hydrophobic surface enrichment. As the amount increases, lignin hydroxyl groups or micro-aggregates may become more visible on the surface, offsetting part of the hydrophobic advantages. Nonetheless, the constant increasing trend suggests that even minor lignin additions can slightly improve the surface hydrophobicity of PVA. Phansamarng et al. [72] obtained similar increased hydrophobicity with less water absorption when up to 5% lignin was used for PVA mixtures.

**Figure 9:**
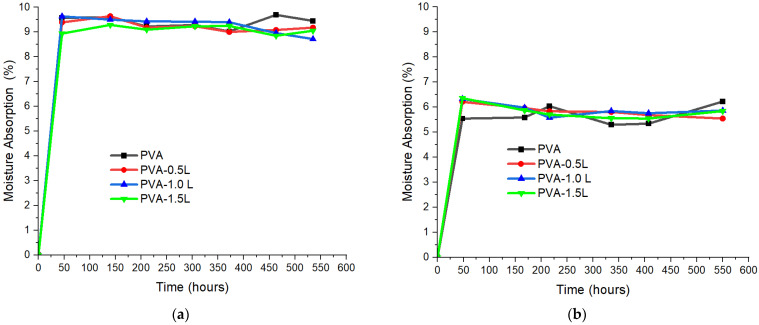
Moisture absorption ratios of PVA and PVA/lignin mixtures at (**a**) 85% and 85 °C and (**b**) 75% and 75 °C conditions.

Figure 9a illustrates how the PVA and PVA/lignin films absorb moisture when exposed to high humidity (85%) and temperature (85 °C) levels for an extended period. All samples showed quick uptake in the first 48 h. While lignin-containing films displayed somewhat lower results, with the 1.5 weight percent lignin sample reaching the lowest equilibrium moisture content (~8.9%), neat PVA achieved an equilibrium moisture content of ~9.5%. The reduction in moisture absorption, while minor (1–6% relative), suggests that lignin inclusion contributes to decreased water affinity and free volume within the polymer network. The hydrogen bonding between lignin and PVA may serve as a crosslink-like interaction, limiting chain mobility and the accessibility of hydroxyl groups to water molecules. The interactions of hydrophobic sites of modified PVA and lignin’s hydrophobic aromatic domains may also have contributed positively to this observed result. Furthermore, dispersed lignin domains can increase the tortuosity of diffusion routes, reducing the penetration of water. Similarly, Ramadhan et al. [53] asserted that PVA/lignin films have enhanced water resistance relative to pure PVA due to the formation of a new layer of long-chain lignin polymer on the surface of the lignin/PVA films. The nonlinear trend across lignin concentrations provides support to the theory that different parameters determine the total moisture response. At lignin concentrations of 0.5 and 1.5 wt%, barrier and hydrogen bonding effects dominate, resulting in decreased absorption. At 1.0 wt%, however, a minor increase was found compared to the 0.5 wt% sample, which could be attributed to local disruption of crystallinity or the creation of micro gaps produced by less uniform dispersion, increasing water accessibility.

Although the observed reduction in moisture uptake is minor, such improvements can nevertheless be useful in multilayer or hybrid barrier systems, where incremental reductions at each layer adds to a considerable overall performance improvement. In particular, when paired with hydrophobic coatings or nanofillers, the presence of lignin within PVA may diminish the tortuosity of the permeability pathway, hence reducing water vapour transfer synergistically. This emphasises the significance of even little changes in moisture resistance, particularly for applications like packaging, where barrier performance is dependent on the cumulative influence of numerous components.

Figure 9b depicts the moisture absorption (%) of pure PVA and PVA/lignin composites over time as exposed to 75% and 75 °C settings. All samples showed rapid initial water uptake within the first 48 h, followed by a plateau showing equilibrium moisture content. This two-stage behaviour is nearly identical to the 85%—85 °C samples (Figure 9a). The results show that pure PVA had a constant equilibrium absorption level of around 6%. However, the addition of lignin changed this behaviour depending on the filler amount. Water absorption at 0.5% lignin was comparable to that of pure PVA, indicating that the low lignin component did not drastically inhibit water penetration. With 1.0% lignin, equilibrium absorption reduced marginally, indicating stronger hydrogen bonding interactions between PVA and lignin, reducing the amount of free hydroxyl groups available for water sorption. Interestingly, at 1.5% lignin, water absorption increased again, approaching values comparable to that of pure PVA. This could be due to lignin agglomeration at higher loadings, which affects the polymer matrix and causes micro voids or interfacial defects that allow water to penetrate, as described in the 85%—85 °C experiments.

Overall, the findings of the two humidity tests indicate that while excessive lignin loading (1.5%) causes structural heterogeneity and increases susceptibility to moisture absorption, intermediate lignin concentrations (1.0%) can increase PVA’s water resistance by strengthening interfacial bonding. Under both circumstances, the addition of lignin somewhat decreased moisture uptake; the impact was more pronounced at lower temperatures and humidity levels, where PVA and lignin’s hydrogen bonding more successfully inhibited water sorption.

According to previous studies, chemical modifications including acetylation, esterification, and phenolation can improve dispersion within PVA films, decrease lignin polarity, and enhance interfacial adhesion with hydrophilic and hydrophobic matrices [18,23]. For example, acetylated lignin has been shown to reduce aggregation and boost mechanical performance in PVA blends, whereas esterified lignin improves hydrophobicity and barrier characteristics [51]. Furthermore, the application of compatibilizers or coupling agents (such as maleic anhydride, silanes, or polyethylene glycol) has been shown to improve filler–matrix bonding, resulting in increased tensile strength and processability. Incorporating such strategies into future research could not only improve adhesion concerns identified at greater lignin loadings, but also reveal higher performance levels, making PVA/lignin composites more practical for commercial applications, notably in the packaging and coating industries.

## 4. Conclusions

This study explored the effects of lignin integration on the rheological, thermal, and moisture absorption properties of polyvinyl alcohol (PVA) films. The findings show that lignin can serve as both a reinforcing and modifying agent, although its effectiveness is highly reliant on concentration. At low to moderate loadings, lignin contributes to stronger polymer–filler connections via hydrogen bonding, increasing the elastic responsiveness of the composite while reducing the availability of hydroxyl groups for water uptake. These interactions also marginally improve thermal stability by increasing char yield, which is an important feature for flame resistance. However, with greater loads, lignin’s tendency to aggregate lowers interfacial efficiency and introduces structural variability, cancelling out some of the benefits seen at lower concentrations.

From an application standpoint, these findings underline lignin’s potential as a sustainable and low-cost additive for optimising the balance of processability, thermal stability, and moisture resistance in PVA-based systems. Importantly, these findings indicate that small amounts of lignin may be sufficient to produce functional improvements while keeping crucial host polymer features such as melt processability.

A potential future direction is to use surface-modified or fractionated lignin to improve compatibility with PVA, minimise aggregation, and extend performance benefits. These can be addressed by modifying the surface of lignin (for example, acetylation, esterification, or phenolation), including compatibilizers, or employing chemical crosslinking techniques. Such methods could promote filler dispersion, strengthen interfacial adhesion, and maximise property improvements. Such approaches could open the way for the development of biodegradable composites ideal for packaging, coatings, and fibre-reinforced constructions with increased durability and lower environmental impact.

Future studies could reduce variabilities by using fractionated lignin with narrower molecular weight distributions or enriched S/G ratios, thereby improving reproducibility of rheological measurements. While the present study focused on thermal, rheological, and moisture properties, future work can include tensile and long-term testing to fully evaluate the mechanical and moisture absorption performance of PVA/lignin composites for different applications. Although the optical microscopy provided preliminary evidence of lignin micro-aggregates, higher-resolution techniques such as SEM, TEM, or AFM would offer more definitive insights into dispersion and interfacial interactions and thus represent an important direction for future work.

## Figures and Tables

**Table 1:** Material content of different mixtures.

Sample Code	PVA Content (%)	Lignin Content (%)
PVA	100	-
PVA-0.5L	99.5	0.5
PVA-1.0L	99.0	1.0
PVA-1.5L	98.50	1.5
PVA-3.0L	97.00	3.0
PVA-5.0L	95.00	5.0
PVA-10L	90.00	10

**Table 2:** DSC results of PVA and PVA/lignin blends.

Samples	1st Heating		Cooling	2nd Heating	
	Tm (°C)	Xc (%)	Tc (°C)	Tm (°C)	Xc (%)
PVA	214.03	48.77	195.07	215.53	46.31
PVA-0.5L	213.91	49.66	194.31	214.45	46.00
PVA-1.0L	214.45	49.61	194.36	215.73	43.01
PVA-1.5L	214.07	48.03	192.96	214.90	45.97

**Table 3:** TGA results for lignin, PVA, and PVA/lignin mixtures.

Samples	Degradation Onset Temperature (°C)	DTGA Peak (°C)	Residue at 700 °C (%)
Lignin	257.9 (±8.3)	378.3 (±0.7)	42.75 (±0.40)
PVA	351.9 (±5.7)	393.0 (±1.6)	0.48 (±0.03)
PVA-0.5L	351.1 (±3.4)	392.5 (±2.6)	0.40 (±0.28)
PVA-1.0L	358.9 (±2.2)	396.4 (±6.5)	1.42 (±0.59)
PVA-1.5L	356.1 (±3.0)	394.4 (±0.5)	1.68 (±0.42)
PVA-3.0L	355.1 (±0.1)	387.7 (±5.6)	1.70 (±0.91)
PVA-5.0L	353.7 (±2.4)	391.3 (±2.7)	2.13 (±0.10)
PVA-10L	352.1 (±0.5)	389.9 (±0.6)	4.15 (±0.10)

## Data Availability

The original contributions presented in this study are included in the article. Further inquiries can be directed to the corresponding author.
